# Salivary Calculi

**Published:** 1865-12

**Authors:** 


### SALIVARY CALCULI.

A patient, 25 years old, entered the Massachusetts General Hospital for a tumor beneath the ramus of the jaw, of twelve years' standing, hard, movable, and from which no inconvenience had been experienced until six months before her admission when it became painful, enlarged, and easily felt within the mouth at the side of the tongue. It discharged a watery fluid at first, which subsequently became purulent. Every now and then the tumor would enlarge, the discharge cease for a short time, and then break out again, with relief. An incision at the side of the tongue permitted the release of two salivary calculi — one the size of a grain of wheat, the other not larger than a mustard-seed. The incision healed rapidly, and three months after the operation the patient was seen, and found to have been relived entirely from the periodical attacks of swelling, while the tumor was rapidly disappearing.—*Boston Med. and Surg. Journal.*

